# Effect of Beverages on Viscoelastic Properties of Resin-Based Dental Composites

**DOI:** 10.3390/ma8062863

**Published:** 2015-05-26

**Authors:** Muhammad Kaleem, Abdul S. Khan, Ihtesham U. Rehman, Ferranti S. Wong

**Affiliations:** 1 Army Medical College, National University of Sciences and Technology, Islamabad 44000, Pakistan; E-Mail: mkaleem-amc@nust.edu.pk; 2 Interdisciplinary Research Centre in Biomedical Materials, COMSATS Institute of Information Technology, Lahore 54000, Pakistan; 3 Centre for Oral Growth and Development, Institute of Dentistry, Barts and the London School of Medicine and Dentistry, Queen Mary University of London, London E1 2AD, UK; E-Mail: f.s.l.wong@qmul.ac.uk; 4 Kroto Research Institute, Department of Materials Science and Engineering, University of Sheffield, Sheffield S3 7HQ, UK; E-Mail: i.u.rehman@sheffield.ac.uk

**Keywords:** dental composites, beverages, viscoelasticity, storage modulus

## Abstract

The viscoelastic properties of three commercially available resin-based composites (Filtek™ P60, Filtek™ Supreme, and Filtek™ Z250; 3M ESPE, Bracknell, UK) were measured to determine the effect of beverages on their storage moduli and damping ratios. Rectangular samples of the three hybrid composites were immersed in three beverages at 37 °C for 1, 7, 30, and 60 days. At each time interval, these samples were subjected to three-point bend tests in temperature mode using a Perkin Elmer DMA7 Dynamic Mechanical Analyzer (Perkin Elmer Corp., Waltham, MA, USA) to measure the storage modulus and damping ratio. The immersion time had significant influence on the viscoelastic property of composites and it was found that generally for all samples the storage modulus was reduced, whereas damping values increased with immersion time. The viscoelastic behavior of tested materials seems to be related to the pH environment, hydrophilicity and the chemical composition of composites.

## 1. Introduction

Resin-based composite (RBC), as a dental restorative material, is heterogeneous and isotropic [1]. It is a polymer-based material, which transforms from a liquid to a rubbery-glassy state on polymerization. When subjected to an erosive condition, material may be lost due to monomers and oligomers leaving the matrix. Factors that may influence the speed of this reaction include the type of material’s chemical bond, copolymer composition, hydrophilicity of the polymer matrix and the pH of the erosive agents [2]. The long-term stability as a restorative material is frequently related to its resistance to degradation under the sinusoidal masticatory load and effect of oral environment, especially with the increasing consumption of beverages [3]. Several researchers [4,5,6,7,8] have investigated the effects of food-simulating liquids on the properties of dental composites, however, to date, the effects of these agents on the viscoelastic properties of the restorative materials have not been tested.

Viscoelastic measurements can provide information on the polymeric system by measuring the propensity of the material to creep under load. Viscoelastic solids differ from elastic solids in that [9]: (i) on application of a constant stress, after the instantaneous strain, the strain increases with time (creep); (ii) on application of a constant strain, after the instantaneous stress, the stress decreases with time (stress relaxation); and (iii) if an alternating (sinusoidal) stress or strain is applied, stress and strain are out of phase. This phase angle is denoted by δ. Theoretically, the Young’s modulus (*E*) of classical elasticity is replaced by the so-called complex modulus (E*), where E* is given by

(1) E^*^ = *E*^′^ + i*E*^″^

where, *E*′ is the storage modulus, and represents the elastic component of deformation; *E*″ is the loss modulus, and represents the viscous (inelastic) component; and i = √−1. These are related to δ:

(2) *tan* δ *= E″/E′*

where, tan δ is a measure of energy loss, sometimes referred to loosely as damping capacity. It is related to the fraction of energy retained, resilience (*R*):

(3) *R= exp (−*π *tan* δ*)*

At the glass transition temperature (*T*_g_), *E*′ decreases dramatically over a short temperature range, *E*″ decreases initially and then increases, and tan δ go through maxima. Beyond *T*_g_, the polymer is in the rubber-like state, where deformation is a function of entropy only.

Dynamic Mechanical Analysis (DMA) is now a standard method in measuring the viscoelastic properties and is suggested as a valuable tool for characterization of viscoelasticity of dental composites to give a better insight into their mechanical behavior at molecular level [10,11,12,13,14,15]. It measures the ratio of the amplitudes of stress to the applied strain, and the phase angle δ, and computes *E*′, *E*″, and tan δ.

The aim of this study was to investigate the viscoelastic properties of three composite restorative materials after a period of storage in various beverages with different pH values. The working hypothesis for this study was that beverages could affect the viscoelastic stability of RBC restorative materials. This hypothesis was based on the assumption that acidic environment could cause breakage of the polymer-resin bonding with consequential change of the viscoelastic properties of the composite.

## 2. Results

**Figure 1:**
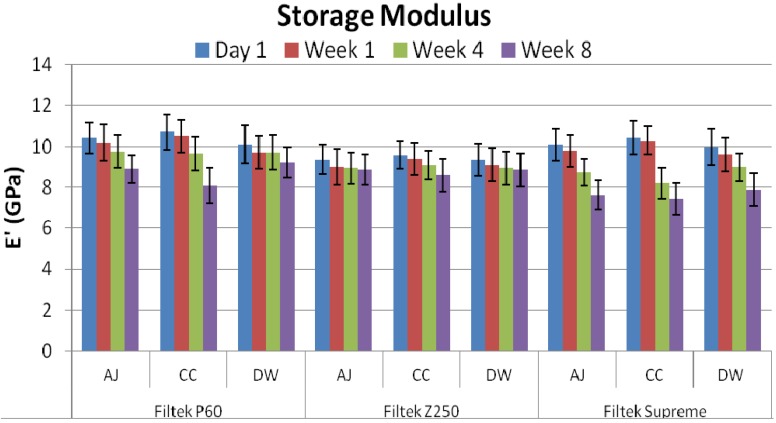
Comparative Storage Modulus values (GPa) of Z250, Filtek™ P60 and Filtek™ Supreme after immersion in apple juice (AJ), Coca-Cola (CC) and deionized water (DW) for one day, one week, four week, and eight week.

**Figure 2:**
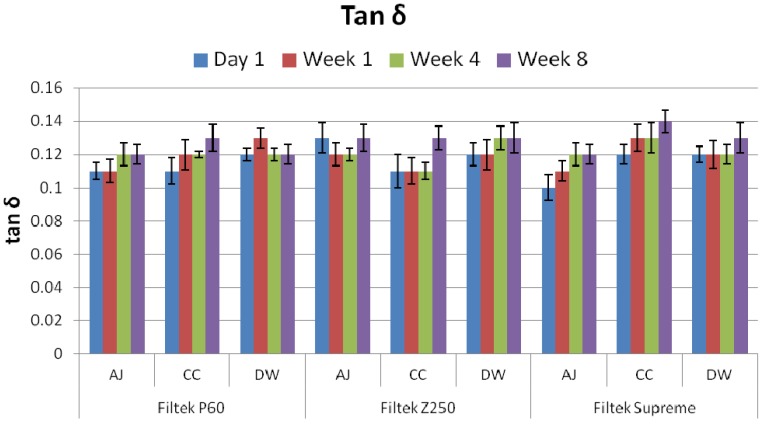
Comparative tan delta values of Z250, Filtek™ P60 and Filtek™ Supreme after immersion in apple juice (AJ), Coca-Cola (CC) and deionized water (DW) for one day, one week, four week and eight week.

The mean storage modulus (*E*′) and mechanical damping (tan δ**)** of all the groups along with their standard deviations are given in Figure 1 and Figure 2, respectively. It was observed that the storage moduli for all samples were decreased with immersion time in media. The statistical analysis indicated that there was no significant difference after immersion for four week, whereas after eight week time period, all three composites showed a significant difference (*p* = 0.037) in all solutions. Samples immersed in apple juice (AJ) and Coca-Cola (CC) showed significant decrease in values as compare to deionized water (DW). Among samples, Filtek™ P60 and Filtek™ Z250 after eight week immersion in AJ, CC and DW showed significant difference (*p* = 0.011) with Filtek™ Supreme, whereas, insignificant difference was observed between Filtek™ P60 and Filtek™ Z250. The tan δ values for composites increased with immersion time, however, the insignificant difference was observed.

## 3. Discussion

The properties of restorative material may degrade in the volatile oral environment. The mean pH of oral cavity is 6.28–7.34 [16]. Many foods and drinks (e.g., water, acidic soft drinks, alcoholic drinks, and food derivatives) affect the behavior of restorative materials. Previous studies have shown that fillers tend to fall out from resin materials [17], and the matrix component decomposes when exposed to low pH environments [18,19]. Many soft drinks are acidic and the pH is 3.0 or lower. This means that drinking acidic drinks over a long period and with continuous sipping can erode the tooth enamel―and the resin material as well. Therefore, the increase in use of RBC restorations made them more prone to be exposed to diverse conditions of oral environment, which can result in chemical degradation [20] and reduction in physical properties [21], subsequently effect on the clinical longevity of the material. There is lack of data about the viscoelastic stability of resin-composite restorative material after being subjected to various daily use beverages.

In the present study, different beverages promoted viscoelastic alterations, which are probably ascribed to a chemical reaction or dissolution of the composite when immersed in the tested beverages. These chemical reactions make the plasticization of polymer matrix and results in partial and complete debonding of filler particles. The variety of the results could be explained by the different testing conditions, especially with respect to storage medium, which has been shown to be important. The degrading effect of water on composites has been explained in the past with two different mechanisms. The first one is the plasticizing role of the water molecule, which creates more volume in the matrix and enhances the movement of the chain segments, resulting in the decrease of the stiffness of the material. The other mechanism is the leaching of composite’s components in the water [22]. The comparative study showed that Filtek™ P60 and Filtek™ Z250 has higher storage modulus compared to Filtek™ Supreme. It could be due to presence of high molecular weight Bisphenol A-glycidyl methacrylate (Bis-GMA), which restricts the molecular motion and decreases the dissipation of energy stored. In Filtek™ P60 and Filtek™ Z250 composites the low molecular weight monomer Triethyleneglycol dimethacrylate (TEGDMA) is replaced by Urethane dimethacrylate (UDMA) and Bisphenol A polyethylene diether dimethacrylate (Bis-EMA), so the reduction in the concentration of TEGDMA leads to higher viscosity. Both of these resins are higher molecular weight and therefore have fewer double bonds per unit of weight. The high molecular weight materials also impact the measurable viscosity and results in less shrinkage, reduced aging and a slightly softer resin matrix. Additionally, these resins impart a greater hydrophobicity and are less sensitive to changes in atmospheric moisture. Filtek™ Supreme composed of BisGMA, UDMA, Bis-EMA and TEGDMA. UDMA has low water absorption and solubility characteristics [23], whereas, TEGDMA is a hydrophilic monomer and able to absorb water [24]. It was found that TEGDMA containing composite showed low storage modulus with immersion time, which was due to increase in water uptake surface hydrophilicity. Hydrophilic groups such as the ethoxy group in TEGDMA are thought to show affinity with water molecule by hydrogen bonding to oxygen [25]. However, their comparative values with other composites were due to presence of UDMA-based monomers that causes low viscosity and low water absorption and successful polymerization with visible light [26,27]. On the basis of this, the specific composition and/or characteristics of RBCs that was used in this study may have favored the specific degradation. In respect to specimens immersed in Coca-Cola (pH 2.4), it was found that immersion media promoted degradation pattern on the composites. It is expected that Coca-Cola might damage the integrity of restorative materials because of presence of phosphoric acid. Previous studies [28,29,30] reported the dissolution of calcium phosphate from the tooth structure due to usage of Coca-Cola.

The DMA three-point bend test was used, therefore, the comparison of the modulus values for RBCs are limited to only in the axial direction. The direction of testing load means that the materials were subjected to tension in the lower surface and compression in the upper surface. The general trend for these composites showed that with increase in immersion time the storage modulus was reduced, whereas damping values increased which indicate that the RBCs absorbs less energy in repeated stress, and the external energy is likely to be dissipated within the material as residual stress. The acidic environment may cause the scission of polymeric chain and due to dynamic force the movement of molecules was expected to increase leading to movement of polymer chain, which reduces the stress and consequently the storage modulus. The force strike at one point and the energy dissipate to adjacent molecules and it enhances the molecular movement. The more the molecular movement, the lower is the stiffness. The pattern of stress distribution in a three-point bend test is similar to the common pattern of stress distribution in mastication and is clinically relevant [31].

Mostly in literature, the modulus values of composites were obtained at room temperature (21 ± 3 °C), whereas, the viscoelastic parameter is temperature dependent. Therefore, in this study the data were obtained at 37 °C, where testing results are very important in order to predict the clinical performance. The standard deviation of the measured values in the present study might be attributed to the variation in the roughness of the specimen surface because all the specimens were prepared manually, and the testing configuration is sensitive to surface flaws and defects. However, this feature appears to be consistent with previous mechanical and thermal studies [32,33,34,35]. It would be of interest to compare the viscoelastic moduli of these materials to those of natural tooth tissue. It has been shown that the relaxation modulus of human dentin has a linear dependence on the logarithm of time [36], indicating that dentin is also a viscoelastic material. The linear dependence of relaxation or creep on log time has been shown by Gent [37] to be applicable for elastomers and Braden and Wilson [38] for glass ionomer cements. The authors were unable to find any report that used DMA to measure the viscoelastic moduli for dentin in order to make direct comparison. The Young’s modulus for dentin, measured by simple bending technique, was reported to be in the range of 11.4–19.3 GPa [39]. This value is lower than the storage modulus for intertubular dentin, 21 GPa (range 17–23 GPa), measured by combining a nano-indentation technique with Atomic Force Microscopy [40].

## 4. Clinical Significance

As these restorative materials have to be placed in the teeth, it is very important that these materials behave in the similar manner as the enamel and dentin, when they were subjected to occlusal forces. Therefore, it could be observed that beverages promoted a significant degradation of the resin matrix, even within the period of evaluation used in the present study, demonstrating that the clinicians must advise patients about the possible effects of these solutions over composites. According to this study, Filtek™ P60 showed better results compared to other restorative materials, however, further studies are necessary to verify the influence of beverages on other mechanical and physical properties.

## 5. Materials and Methods

Three different types of commercial photo-activated resin-based composite (RBC) restorative materials (Filtek™ P60, Filtek™ Supreme and Filtek™ Z250, 3M ESPE, Bracknell, UK) were used. The resin system and filler particle size with their coloring shades are given in Table 1. The resin matrices in these commercial products are BIS-GMA, BIS-EMA, UDMA, where, in Filtek™ Supreme there is small amount of TEGDMA along with UDMA. In Filtek™ Supreme, the filler contains a combination of a non-agglomerated/non-aggregated, 20 nm nano-silica filler, and loosely bound agglomerated zirconia/silica nano-cluster, consisting of agglomerates of primary zirconia/silica particles with size of 5–20 nm fillers. The cluster particle size range is 0.6 to 1.4 microns. The filler loading is 78.5% by weight. In Filtek™ P60 and Filtek™ Z250, the particle size distribution of filler is 0.01 µm to 3.5 µm with an average particle size of 0.6 µm. The fillers are zirconia/silica with 61% and 60% by volume without silane treatment, respectively.

**Table 1:** Composition of commercial dental composites with filler particle size and loading by volume.

Material	Shade	Fillers Particle size/Loading by volume	Resin System Ϯ
Filtek™ P60 Batch No. 4720	A3	0.01–3.5 µm/61% vol.	Bis-GMA, UDMA and Bis-EMA
Filtek™ Z250 Batch No. 6020	A2	0.01–3.5 µm/60% vol.	
Filtek™ Supreme Batch No. 3910	A2B	5–75 nm/57%–60% vol.	Bis-GMA, Bis-EMA and TEGDMA

Notes: Ϯ Resin System: Bis-GMA = Bisphenol A-glycidyl methacrylate; UDMA = Urethane dimethacrylate; BisEMA = Bisphenol A polyethylene diether dimethacrylate; TEGDMA = Triethyleneglycol dimethacrylate.

### 5.1. Sample Preparation

The RBCs were used without medication and were handled according to the manufacturer’s instruction. The composite materials were injected in low light condition directly into PTFE molds (24 × 3 × 1.5 mm^3^) with no bonding agents. The specimens were polymerized by using a blue visible light (ESPE Elipar^®^ Trilight, Seefeld Bavaria, Germany, wavelength ≈ 470 nm). As the sample length was 24 mm, in order to obtain maximum polymerization, curing was carried out in sections and each section was cured for 60 s. The distance of blue visible light was constant (1 mm) for all samples.

To evaluate the effect of liquid diets on these materials, samples were immersed in two experimental media, Coca-Cola (Coke) and Apple Juice (Nestle), and a control medium, deionized water. The composition and pH values of these media are given in Table 2. Ninety specimens were prepared for each composite material. Ten specimens of each material were immersed in each media at 37 °C for 1, 7, 30, and 60 days (*i.e.*, day 1, week 1, week 4 and week 8). The media were changed daily to maintain constant pH. At each time period, the samples were dried and their viscoelastic properties were measures using DMA.

**Table 2:** Solvent used in the study along with contents and pH values.

Solution	Content	pH
Coca-Cola	Carbonated Water Sugar Carbohydrate Phosphoric Acid Caffeine	2.5
Apple Juice	Pure Apple juice (nothing added or removed, no flavor and sugar)	4.0
Deionized Water		7.4

### 5.2. Dynamic Mechanical Analysis

A Perkin Elmer DMA7 (Perkin Elmer Corp., Waltham, MA, USA) in three-point bending mode was used to measure the viscoelastic properties of the materials. For a specimen of known geometry, if *L* = distance between the two supports, *b* = width, and *t* = depth, the oscillating strain (ε_o_) is given by,

(4) ε_o_*= 3ty*^0^*/L*_2_

where, *y*^0^ is the displacement amplitude. The maximum oscillating stress (σ_o_) occurs on the upper and lower surfaces and was given by,

(5) σ_o_*= 3F*_0_*L/2bt*^2^

where, *F*_0_ is the axial force amplitude. Therefore, by substituting for stress and strain, the complex modulus (E*) was given by,

(6) E^*^ = F_0_*L*^3^*/2y*^0^*bt*^3^

The support separation in three-point bend test was 20 mm and the specimen length was about 24 mm. Prior to testing, the exact dimensions (width and depth) for each specimen were measured at three different points and averaged. Testing was performed in the temperature scan mode using the parameters and conditions shown in Table 3. The temperature was measured with a thermocouple positioned approximately 1 mm away from the sample. Helium gas at a rate of 30 mL min^−1^ was used in the furnace and cooling water maintained the isothermal environment outside the furnace. The measured data were automatically saved at the end of each test using the Pyris Manager software. One way ANOVA statistical test was used to check the significance level between samples for each time period. A *p* value ≤0.05 was regarded as being significant.

**Table 3:** The DMA parameters used in temperature mode.

Parameter	Conditions
Initial Temperature	26 °C
Final Temperature	50 °C
Static Force	400 mN
Dynamic Force	380 mN
Frequency	1 Hz
Heating Rate	10 °C/min
